# Cu/Zn-superoxide dismutase naturally fused with a β-propeller lactonase in *Deinococcus radiodurans*

**DOI:** 10.1016/j.jbc.2025.110499

**Published:** 2025-07-18

**Authors:** Yoshiaki Furukawa, Masamichi Megata, Atsuko Shintani, Kaori Sue, Tomohiro Morohoshi, Masato Akutsu, Norifumi Muraki

**Affiliations:** 1 Department of Chemistry, Keio University, Yokohama, Kanagawa, Japan; 2 Graduate School of Regional Development and Creativity, Utsunomiya University, Utsunomiya, Tochigi, Japan; 3 Research Center for Advanced Analysis, National Agriculture and Food Research Organization (NARO), Tsukuba, Ibaraki, Japan

**Keywords:** superoxide dismutase (SOD), multifunctional enzyme, calcium-binding protein, crystal structure, hydrolase

## Abstract

Cu/Zn-superoxide dismutase (Cu/Zn-SOD) is an antioxidant enzyme widely present across species; however, the structural diversity and physiological roles of Cu/Zn-SOD are yet to be fully uncovered. Here, we show a unique type of Cu/Zn-SOD from *Deinococcus radiodurans* (DrSOD) with an additional β-propeller domain. Our structural analysis of DrSOD revealed a typical bacterial Cu/Zn-SOD domain, binding both a copper and zinc ion, alongside a six-bladed β-propeller domain coordinating a calcium ion. DrSOD was indeed expressed in *D. radiodurans*, but its deletion did not lead to any noticeable changes in resistance to DNA-damaging stresses, a characteristic trait of *D. radiodurans*. Despite this, the Cu/Zn-SOD domain retained superoxide dismutase activity, and the β-propeller domain was found to exhibit a lactonase activity specifically for hydrolyzing 2-coumaranone. Taken together, while the precise physiological role of DrSOD needs to be further investigated, our findings here reveal a unique multi-functional enzyme architecture, expanding the known structural diversity of Cu/Zn-SODs.

Cu/Zn-superoxide dismutase (Cu/Zn-SOD) is an antioxidant enzyme found across a wide range of organisms, where it plays a role in detoxifying superoxide radicals by catalyzing their dismutation into molecular oxygen and hydrogen peroxide (1). Structural and functional properties of Cu/Zn-SOD from bacteria to mammals are important for understanding various pathological as well as physiological processes, such as infection, energy metabolism, and neurodegenerative diseases (2, 3).

The basic structure of Cu/Zn-SOD is characterized by an immunoglobulin-like fold consisting of eight β-strands arranged in a Greek-key β-barrel configuration (4). Additionally, a copper ion serves as the catalytic center, and a zinc ion is bound at a distinct site to maintain the catalytically competent conformation (4). Depending on the species or isoform, Cu/Zn-SOD can exist as a monomer, homo-dimer, or homo-tetramer, with several distinct arrangements of subunits observed (3). Consequently, proteins classified as Cu/Zn-SOD exhibit remarkable structural diversity in their configurations despite highly conserved subunits.

Furthermore, the Cu/Zn-SOD subunit can act as a domain within multi-domain proteins, potentially contributing to unique structural and/or enzymatic functions. For example, the Cu/Zn-SOD-like domain, despite lacking a copper-binding site and thus enzymatic activity, plays a critical role as a component of the copper chaperone for superoxide dismutase (CCS), specifically recognizing Cu/Zn-SOD and enabling the precise delivery of the copper ion required for its activation (5, 6). Additionally, we have previously reported that a Cu/Zn-SOD-like protein from *Paenibacillus lautus* is composed of the Cu/Zn-SOD domain and an authentic dimerization domain (7). Notably, 276 domain architectures containing the Cu/Zn-SOD domain are currently registered under the entry IPR001424 in InterPro (8), a comprehensive resource for protein family classification provided by EMBL-EBI. Accordingly, Cu/Zn-SOD is presumed to play important roles as a domain within multi-domain proteins.

Among those multi-domain proteins, we recently noted a unique protein in which a Cu/Zn-SOD domain is fused with an SMP-30/Gluconolactonase/LRE-like region (SGL) domain characterized by a six-bladed β-propeller structure. Interestingly, all 10 proteins with this domain architecture (IPR001424 with IPR013658 in the InterPro entry) are found exclusively in the *Deinococcus* genus, known for its exceptional resistance to extreme environmental conditions (9). In *Deinococcus radiodurans*, a representative species of *Deinococcus*, the unique Cu/Zn-SOD fused with the SGL domain is registered as DRA0202 (referred to hereafter as DrSOD), which is one of four distinct superoxide dismutases encoded in *D. radiodurans*. It is well known that cytoplasmic manganese superoxide dismutase (DR1279) plays a crucial role in supporting the survival of *Deinococcus* under ionizing radiation (10, 11); however, the other three superoxide dismutases, including DrSOD, remain largely unexplored.

Each of the three Cu/Zn-SOD proteins in *D. radiodurans* possesses an N-terminal signal peptide, consistent with their predicted localization in the periplasmic space. DR1546 (referred to hereafter as DrSodC) shares approximately 40% sequence similarity with *Escherichia coli* Cu/Zn-SOD (EcSodC) and also exhibits a highly similar tertiary structure predicted by ColabFold (12), suggesting that it likely functions as a canonical Cu/Zn-SOD in *D. radiodurans*. DrSodC has an N-terminal cysteine residue immediately following signal peptide cleavage, which is likely lipidated and may serve to anchor the protein to the membrane. DR0644 lacks one of the histidine ligands required for copper binding, the catalytically essential arginine residue, and a significant portion of the zinc-binding site, raising questions about its capacity to act as a superoxide dismutase. Interestingly, a recent study revealed that DR0644 forms a trimeric structure surrounding the S-layer Deinoxanthin-binding complex contains the carotenoid-binding protein SlpA and is embedded in the cell envelope (13). This complex binds the antioxidant pigment deinoxanthin, which quenches reactive oxygen species and contributes to oxidative stress resistance in *D. radiodurans* (14). The collar-like arrangement of DR0644 suggests a possible role in supporting this chemoprotective system (13). In *D. radiodurans*, manganese superoxide dismutase (DR1279) is constitutively expressed and is thought to play a central role in superoxide detoxification; in contrast, Cu/Zn-SOD proteins, including DrSOD and DrSodC, are reported to be expressed only under a few specific culture conditions and at relatively low abundance (15). The structural and functional characteristics of DrSOD, including its potential role as superoxide dismutase, remain to be investigated.

In this study, we revealed a unique structure of DrSOD, where the Cu/Zn-SOD domain (DrSOD^SOD^) is fused with a six-bladed β-propeller domain (DrSOD^β-pro^). As expected, DrSOD exhibited superoxide dismutase activity through its DrSOD^SOD^, which was comparable to that of EcSodC, but relatively lower than that of human Cu/Zn-SOD (SOD1). Notably, we found that DrSOD exhibited lactonase activity in its DrSOD^β-pro^, catalyzed by a calcium ion bound at the active site, with high substrate specificity for 2-coumaranone. We confirmed the expression of DrSOD in *D. radiodurans* during the stationary phase, although its physiological role in this microorganism remains unresolved. Nevertheless, our findings highlight the structural versatility of Cu/Zn-SOD, suggesting a potential evolutionary strategy for integrating antioxidant activity with distinct enzymatic functions.

## Results and discussion

### DrSOD is expressed in *D. radiodurans* at the stationary phase

**Figure 1:**
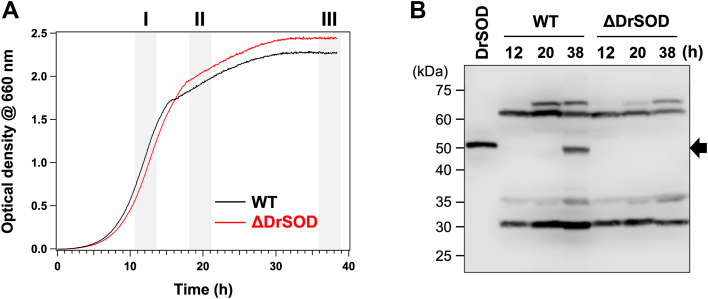
**Expression of DrSOD in *D. radiodurans*.***A*, growth curves of wild-type (WT) and ΔDrSOD *D. radiodurans* cultured in TGY media at 30 °C were monitored by measuring the optical density at 660 nm and are shown in black and red lines, respectively. Samples were collected at three different growth phases: exponential (∼12 h), early stationary (∼20 h), and late stationary (∼38 h), indicated as I, II, and III, respectively, to examine the expression of DrSOD by the Western blot analysis. *B*, Western blot analysis of the expression of DrSOD in WT and ΔDrSOD *D. radiodurans* collected at 12, 20, and 38 h, as described in (*A*). Equal amounts of total protein (50 μg) were loaded in each lane. The appearance of the band corresponding to DrSOD in the late stationary phase of WT but not ΔDrSOD *D. radiodurans* was consistently observed in more than three independent experiments. Recombinant DrSOD protein (labeled as DrSOD, 10 ng) was included as a positive control.

To determine whether DrSOD is expressed in *D. radiodurans*, a strain lacking the gene encoding DrSOD (ΔDrSOD) was generated. As shown in Figure 1*A*, the growth curve of ΔDrSOD was comparable to that of wild-type *D. radiodurans* (WT). WT and ΔDrSOD cells were harvested during the exponential phase, early stationary phase, and late stationary phase (labeled as I, II, and III in Fig. 1*A*), and their lysates were subjected to Western blot analysis using a polyclonal antibody against DrSOD. As shown in Figure 1*B,* a band corresponding to DrSOD was detected in the lysate of WT specifically during the late stationary phase, while no such band was observed in the lysates of ΔDrSOD. These findings confirm that DrSOD is indeed expressed in *D. radiodurans* and is specifically produced during the late stationary phase.

*D. radiodurans* is renowned for its exceptional resistance to DNA-damaging stresses, including ultraviolet (UV) irradiation and exposure to the DNA crosslinking agent mitomycin C (9). To investigate the potential role of DrSOD in those stress responses, we examined the survival of WT and ΔDrSOD following exposure to UV light (254 nm) or mitomycin C. Upon irradiation with 300 mJ of UV light, both WT and ΔDrSOD reduced their growth but exhibited comparable survival (Fig. S1*A*), confirming the intrinsic UV tolerance of *D. radiodurans* while indicating that DrSOD does not contribute to this resistance. Another periplasmic Cu/Zn-SOD, DrSodC, was considered a potential compensator for the role of DrSOD in UV tolerance in *D. radiodurans*; however, deletion of DrSodC alone or in combination with DrSOD (ΔDrSodC and ΔDrSOD/ΔDrSodC) did not affect bacterial growth following UV exposure (Fig. S1*A*). Similarly, treatment with 10 μg/ml mitomycin C led to extensive cell death in *D. radiodurans*, yet no significant difference in survival was observed between WT and ΔDrSOD (Fig. S1*B*). These findings suggest that DrSOD is not involved in the well-characterized DNA damage resistance mechanisms of *D. radiodurans*. Although DrSOD is specifically expressed during the stationary phase, its physiological role remains unclear and warrants further investigation.

### DrSOD is expected as a multidomain protein

**Figure 2:**
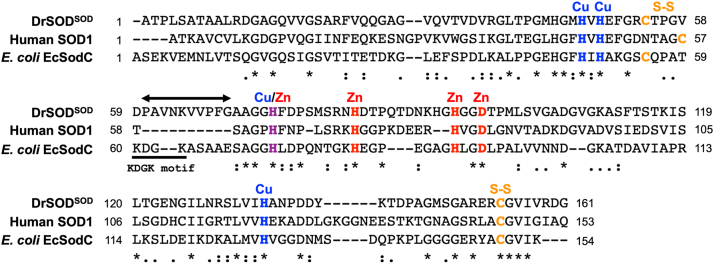
**The sequence alignment of DrSOD^SOD^ with human SOD1 and *E. coli* EcSodC**. The amino acid sequence of DrSOD^SOD^ was aligned with that of human SOD1 and *E. coli* EcSodC by using CLUSTALW. Identical residues are marked with an *asterisk* (∗), conserved residues with similar properties are shown by a *colon* (:), and partially conserved residues by a *dot* (.). Residues involved in copper and zinc binding are highlighted in *blue* and *red*, respectively. Cys residues responsible for the disulfide bond are marked in *orange*. Double-headed *arrows* indicate the extended S-S subloop, a characteristic structural feature of prokaryotic Cu/Zn-SOD proteins. The KDGK motif in the S-S subloop of *E. coli* EcSodC is also indicated.

Registered as Q9RYV4 in UniProt, DrSOD consists of 462 amino acids, with the N-terminal 23 amino acids identified as a signal peptide. As described in the Experimental procedures, the protein excluding the signal peptide is referred to as DrSOD hereafter. According to the AlphaFold Protein Structure Database (EMBL-EBI), the N-terminal region following the signal peptide corresponds to the Cu/Zn-SOD domain (Ala24–Gly184, referred to as DrSOD^SOD^). DrSOD^SOD^ shares significant similarity (approximately 50% similarity and 30% identity) in its primary sequence with Cu/Zn-SOD from *E. coli* -EcSodC and human SOD1. As shown in Figure 2, furthermore, the amino acid residues responsible for binding a copper and zinc ion, as well as the cysteine residues forming the disulfide bond, are conserved. Notably, DrSOD^SOD^ features an insertion of 10 amino acid residues relative to human SOD1 (Fig. 2, double arrow), corresponding to an extended S-S subloop unique to prokaryotic Cu/Zn-SOD. Indeed, this insertion is not apparent when compared to EcSodC (Fig. 2). Thus, DrSOD^SOD^ is considered to be classified as a prokaryotic Cu/Zn-SOD.

Following DrSOD^SOD^, the predicted structure of DrSOD reveals a β-propeller fold with six blades in the C-terminal region (DrSOD^β-pro^, Leu185–Phe462), categorized under the SGL family (IPR013658 in InterPro). DrSOD^β-pro^ is also registered as a sugar lactone lactonase, YvrE, in the NCBI database (GenBank accession number: AAF12178.1); however, sequence alignment of DrSOD^β-pro^ with *Bacillus subtilis* YvrE and other representative proteins from this family, including XC5397 from *Xanthomonas campestris*, Drp35 from *Staphylococcus aureus*, and SMP-30 from human, indicates limited similarity in the primary sequence, with approximately 30% similarity and 15% identity. Despite their shared characteristic of a six-bladed β-propeller structure, they are highly diversified in both sequence and substrate specificity; therefore, we sought to experimentally elucidate the structure and function of DrSOD.

### DrSOD^SOD^ shares typical structural features with prokaryotic Cu/Zn-SOD

**Figure 3:**
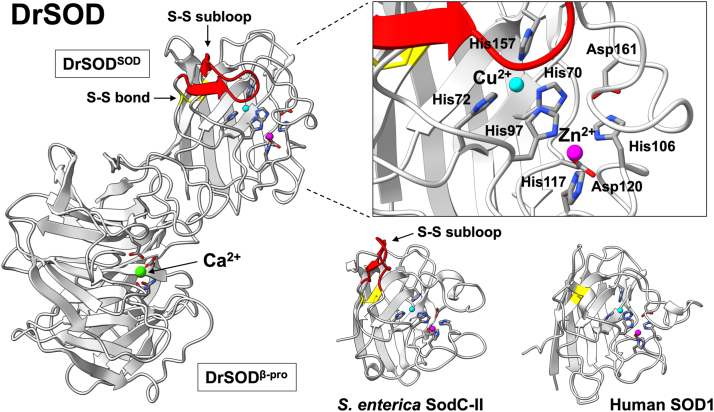
**Crystal structure of DrSOD bound to a copper, zinc, and calcium ion.** The overall structure of DrSOD is depicted as a cartoon model on the left. A copper, zinc, and calcium ion are represented in *cyan*, *magenta*, and *light green*, respectively, with their coordinating ligands shown as stick models. In DrSOD^SOD^, the disulfide bond is highlighted in *yellow*, and the S-S subloop is colored *red*. The *top right panel* shows a magnified view of the copper and zinc binding site in DrSOD^SOD^. Additionally, the overall structure of DrSOD^SOD^, including the copper and zinc binding site and the S-S subloop, is compared with that of *S. enterica* SodC-II (PDB ID: 2K4W) and human SOD1 (PDB ID: 1HL5) in the *lower right panel*.

Figure 3 presents the crystal structure of DrSOD, confirming its multi-domain architecture consisting of an N-terminal Cu/Zn-SOD domain (DrSOD^SOD^) and a C-terminal β-barrel domain (DrSOD^β-pro^). Notably, the overall structure of this experimentally determined crystal structure showed an excellent agreement with the one registered in the AlphaFold Protein Structure Database (EMBL-EBI), as evidenced by a low RMSD value of 1.159 Å between the two models.

DrSOD^SOD^ was shown to bind a copper and zinc ion, as determined using anomalous scattering data collected at wavelengths of 1.373 Å (specific for copper) and 1.275 Å (for both copper and zinc) (Fig. S2). The coordination structure of the copper and zinc ion, a hallmark of Cu/Zn-SOD proteins, is well conserved: the copper ion is coordinated by His70, His72, His97, and His157, while the zinc ion is coordinated by His97, His106, His117, and Asp120 (Fig. 3, top right). His97 serves as a bridging ligand between the copper and the zinc ion, and Asp161 functions as a secondary bridge by forming hydrogen-bonding interactions with His70 and His106—both of which are defining features commonly observed in Cu/Zn-SOD proteins.

The overall fold of DrSOD^SOD^ is highly conserved among Cu/Zn-SOD proteins, characterized by eight β-strands arranged in a Greek key β-barrel configuration and by an intramolecular disulfide bond between Cys77 and Cys177. DrSOD^SOD^ forms the extended S-S subloop, which is a structural feature in prokaryotic Cu/Zn-SOD proteins and is not evident in the eukaryotic counterpart (Fig. 3). Figure 3 also illustrates the conformation of the S-S subloop in DrSOD^SOD^ compared to that in *Salmonella enterica* SodC-II as a representative prokaryotic Cu/Zn-SOD. Notably, the subloop in DrSOD^SOD^ (Pro79–Pro90), which is colored red in the structure shown left in Figure 3, adopts a distinct conformation that appears to act as a lid over the copper-binding site, whereas the subloop in *S. enterica* SodC-II (Pro57-Ala67) displays a more extruded configuration (the structure shown bottom right in Fig. 3). There is a caveat, however, that the subloop’s conformation may be influenced by potential steric obstruction from DrSOD^β-pro^ in the packing of the DrSOD crystal structure.

The subloop in DrSOD^SOD^ is also unique in its amino acid sequence, as it lacks the conserved KDGK motif (Fig. 2), which is critical for the electrostatic recognition and guidance of the substrate, O_2_^-^, by typical prokaryotic Cu/Zn-SOD. Instead, the subloop in DrSOD^SOD^ appears to exhibit a predominantly hydrophobic character (Fig. 2). Actually, the catalytic efficiency of DrSOD^SOD^ as the Cu/Zn-SOD enzyme was relatively low (see below), which might be due to the S-S subloop lacking the KDGK motif. Furthermore, the mutant DrSOD^SOD^, in which the subloop was removed by deleting the region between Pro79 and Pro90, was expressed as inclusion bodies in *E. coli* and proved challenging to refold into a soluble protein (data not shown). These observations suggest that the subloop plays an essential role in maintaining structural integrity as well as affecting the catalytic efficiency of DrSOD^SOD^.

### DrSOD^β-pro^ is a six-bladed β-propeller protein with a calcium ion

**Figure 4:**
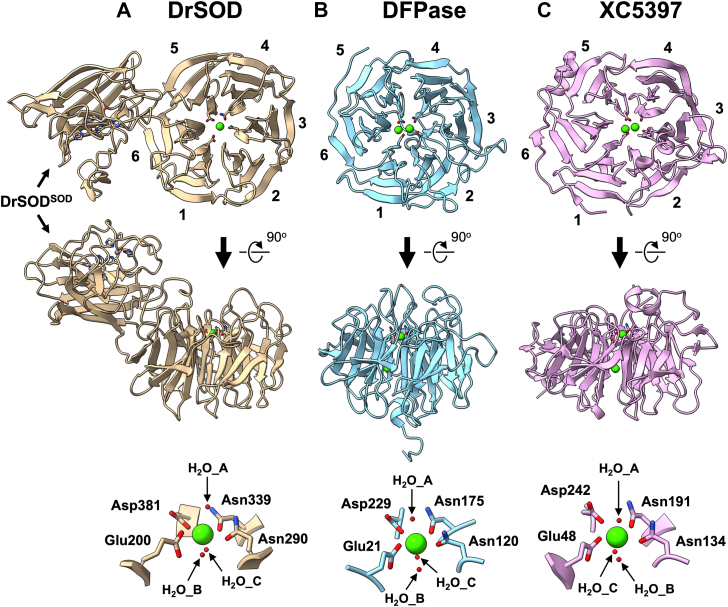
**Structural comparison of DrSOD^β-pro^ with 6-bladed β-propeller proteins** The structure of (*A*) DrSOD^β-pro^ is aligned with that of (*B*) DFPase (PDB ID: 1E1A) and (*C*) XC5397 (PDB ID: 3DR2). The blades forming the β-propeller are numbered sequentially from the N-terminus to the C-terminus. Calcium ions are shown in *light green*, with coordinating ligands depicted as stick models. The figure also includes a view rotated 90 degrees about a horizontal axis. Close-up views of the calcium-binding sites in each protein are presented for detailed comparison.

As predicted in the AlphaFold Protein Structure Database, DrSOD^β-pro^ is confirmed to adopt a β-propeller fold with six blades (Fig. 4*A*, top and middle). Notably, a calcium ion was identified at the center of the β-propeller, coordinated by the oxygen atoms of Glu200, Asn290, Asn339, and Asp381, forming a square planar arrangement (Fig. 4*A*, bottom). Additionally, three water molecules were positioned perpendicular to the plane, completing the first coordination sphere, with two located below the plane and one above it (Fig. 4*A*, bottom). Nonetheless, it should be noted that the electron density maps around one of the ligands for the calcium ion, Asn339, show at least two distinct orientations of the side chain in which the amide group of Asn339 points toward and away from the calcium ion (Fig. S3*A*, bottom). This might represent a weak coordination of Asn339 to the calcium ion, the coexistence of both calcium-bound and -free forms of DrSOD in the crystal, or both.

To elucidate the structure of calcium-free DrSOD, we also analyzed the DrSOD crystallized in the presence of equimolar copper and zinc ions but without the addition of calcium ions. Under these conditions, crystallization occurred at a relatively acidic pH (pH 4.0), which likely hindered the zinc binding (Fig. S3*B*, top). Also, the zinc-binding loop, electrostatic loop, and S-S subloop in DrSOD^SOD^ were not determined, possibly due to significant structural fluctuations (Fig. S3*B*, top). Nevertheless, the overall structure of calcium-free DrSOD crystallized under acidic conditions was nearly superimposable with that of the calcium-bound form crystallized at pH 8.0, with an RMSD of 0.305 Å between calcium-free and calcium-bound DrSOD in chain A. Both crystal structures contain two molecules in the asymmetric unit with no significant differences between them; therefore, the comparison was focused on chain A in both structures. The side chain of Asp339 in the calcium-free DrSOD adopted an orientation away from the center of the β-propeller, resembling the orientation away from the calcium ion in the calcium-bound form of DrSOD (Fig. S3, bottom). While it remains uncertain whether the crystals obtained in the presence of calcium ions contain a mixture of calcium-bound and calcium-free DrSOD molecules, the orientation of the Asp339 side chain pointing toward the calcium ion was exclusively observed in the calcium-bound form. These observations thus suggest that Asp339 plays a role in the binding of the calcium ion.

Among calcium-binding β-propeller proteins with six blades, distinct types of calcium-binding sites exist, characterized by coordination involving three, four, or five oxygen atoms from Glu, Asp, Gln, or Asn residues (16). In this context, the coordination structure surrounding the calcium ion in DrSOD^β-pro^ closely resembles those observed in diisopropyl fluorophosphatase (DFPase) from the squid *Loligo vulgaris* and gluconolactonase XC5397 from *X. campestris*, including the three water molecules around the calcium ion (Fig. 4, *B* and *C*).

### DrSOD is a monomeric protein in solution

**Figure 5:**
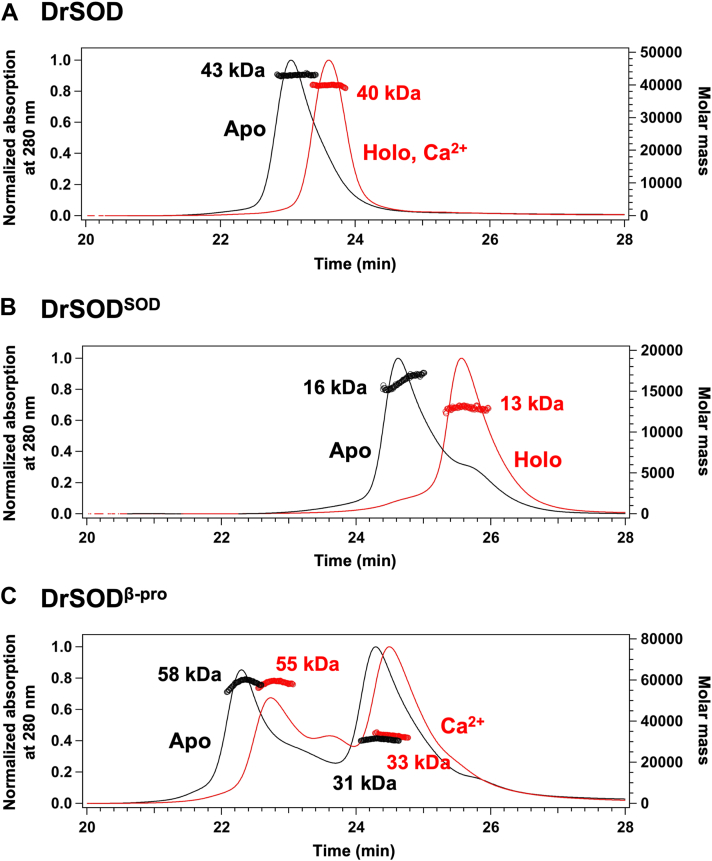
**Analysis of quaternary structures of DrSOD and its individual domains.** SEC-MALS was performed to analyze the quaternary structures of (*A*) full-length DrSOD, (*B*) DrSOD^SOD^, and (*C*) DrSOD^β-pro^ at a concentration of 25 μM. Chromatograms for the metal-free apo state, obtained in the MN buffer containing 5 mM EDTA, are shown as *black lines*. In contrast, chromatograms for the holo forms—(*A*) full-length DrSOD bound to a copper, zinc and calcium ion, (*B*) DrSOD^SOD^ bound to a copper and zinc ion, and (*C*) DrSOD^β-pro^ bound to a calcium ion—were obtained in the MN buffer and are represented as *red lines*. Molecular mass values calculated from MALS analysis are also indicated.

To elucidate the quaternary structure of DrSOD in solution, SEC-MALS analysis was conducted. In the crystal structure of DrSOD, the asymmetric unit comprises two DrSOD molecules. In solution, however, DrSOD in the holo state, where a copper, zinc, and calcium ion are bound, eluted as a single peak with a molar mass of approximately 40,000, closely matching the calculated molar mass (∼45,000) (Fig. 5*A*). This observation indicates that DrSOD exists as a monomer in the holo state. Similarly, DrSOD in the apo state, devoid of bound metal ions, was also eluted as a monomer, albeit with an earlier retention time compared to the holo state (Fig. 5*A*). This shift suggests that metal ion binding induces a conformational change in DrSOD, leading to a more compact structure.

Further analyses of the individual domains revealed that DrSOD^SOD^ also elutes as a monomer in both the apo and the holo state (Fig. 5*B*). As with full-length DrSOD, the apo state exhibited an earlier retention time, indicating a more compact conformation upon binding copper and zinc ions. In contrast, DrSOD^β-pro^ demonstrated a propensity for dimerization, eluting as two distinct peaks corresponding to the calculated molar masses of the monomer (∼30,000) and dimer (∼60,000) (Fig. 5*C*). Again, the retention time of DrSOD^β-pro^ was increased upon binding the calcium ion, suggesting conformational compaction. Collectively, those findings demonstrate that DrSOD is a monomeric protein both in the presence and in the absence of the metal ions and that the metal binding at DrSOD^SOD^ and DrSOD^β-pro^ contributes to a more compact conformation of DrSOD.

### DrSOD exhibits the enzymatic activity as superoxide dismutase

**Figure 6:**
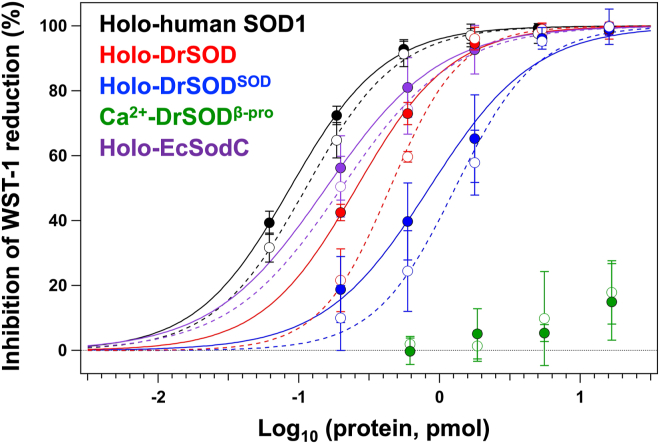
**Assay of superoxide dismutase activity of DrSOD and its individual domains.** The inhibition efficiency of WST-1 reduction was plotted against the amount of each protein in the absence (*filled circles*) and presence (*open circles*) of 1 mM EDTA: holo-SOD1 containing a copper and zinc ion (*black*), holo-DrSOD containing a copper, zinc, and calcium ion (*red*), holo-DrSOD^SOD^ containing a copper and zinc ion (*blue*), DrSOD^β-pro^ containing a calcium ion (*green*), and holo-EcSodC containing a copper and zinc ion (*purple*). The IC_50_ values representing the superoxide dismutase activity were calculated by fitting the data to a sigmoidal function, shown as *dotted lines* for measurements in the presence of 1 mM EDTA and *solid lines* for those in its absence. Each experiment was performed in triplicate, and error bars indicate the standard deviation.

To evaluate whether DrSOD functions as a *bona fide* Cu/Zn-SOD, its superoxide dismutase activity was measured, represented by the amount of protein required to achieve 50% inhibition of the WST-1 reduction by superoxide (IC_50_) (17). As shown in Figure 6, DrSOD in its holo state exhibited significant activity with an IC_50_ of 0.25 ± 0.01 pmol. This activity was slightly lower than that of a representative eukaryotic Cu/Zn-SOD, human SOD1 (IC_50_ = 0.09 ± 0.01 pmol), and comparable to that of a representative prokaryotic Cu/Zn-SOD, *E. coli* EcSodC (IC_50_ = 0.17 ± 0.07 pmol). Although SOD1 is a homodimer while DrSOD and EcSodC are monomeric, IC_50_ values were calculated based on the monomeric units of each protein. It was also confirmed that DrSOD^SOD^, but not DrSOD^β-pro^, exhibited the activity (IC_50_ = 0.90 ± 0.44 pmol), albeit with slightly reduced efficiency compared to full-length DrSOD (Fig. 6). Cu/Zn-SOD is known to bind the metal ions with such high affinity that its enzymatic activity remains largely unaffected in the presence of EDTA, a strong chelator for divalent metal ions. Consistently, the activity of SOD1, EcSodC, DrSOD, and DrSOD^SOD^ was largely preserved even after the addition of 1 mM EDTA, with IC_50_ values of 0.11 ± 0.01, 0.23 ± 0.06, 0.44 ± 0.06, and 1.30 ± 0.48 pmol, respectively (Fig. 6). In addition to copper and zinc binding, the formation of an intramolecular disulfide bond is also essential for superoxide dismutase activity (18, 19). As observed in SOD1 and EcSodC, DrSOD and DrSOD^SOD^ were shown to form a disulfide bond between Cys77 and Cys177, based on the characteristic increase in electrophoretic mobility observed in SDS-PAGE (Fig. S4). Taken together, these results confirm that DrSOD functions as a Cu/Zn-SOD, with its activity attributed to DrSOD^SOD^ and retained even in the presence of EDTA.

### DrSOD exhibits a lactonase activity

As described, our structural analysis revealed that the Ca^2+^-binding site in DrSOD^β-pro^ closely resembles that of DFPase and XC5397 (Fig. 4), raising the possibility that DrSOD may function as a phosphotriesterase and/or gluconolactonase. Based upon the genome analysis using Operon-mapper (20), furthermore, the DrSOD gene forms an operon with its immediately upstream gene encoding a glucose dehydrogenase, although the stop codon of the upstream gene (TGA) overlaps with the start codon of DrSOD (ATG) by sharing the nucleotide A, resulting in a single-nucleotide offset within the operon.

**Figure 7:**
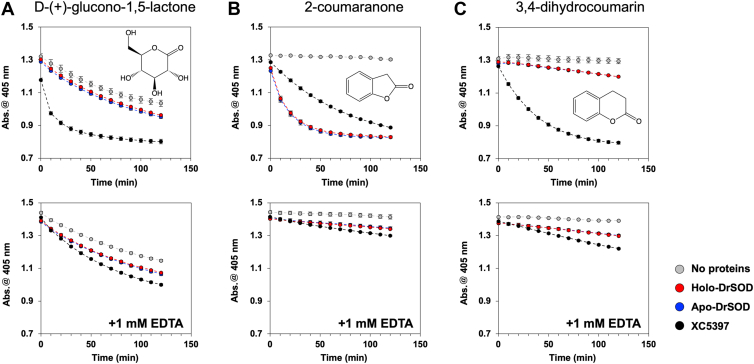
**Assay of lactonase activity of DrSOD**. The absorbance change at 405 nm, reflecting the pH changes of the solution, was monitored at an interval of 10 min using (*A*) D-(+)-glucono-1,5-lactone, (*B*) 2-coumaranone, and (*C*) 3,4-dihydrocoumarin as substrates. Lactonase activity was assessed for (*red*) holo-DrSOD with a copper, zinc, and calcium ion, and (*blue*) apo-DrSOD containing only a calcium without a copper and zinc ion. Negative control without any protein was also included (*gray*). For comparison, the lactonase activity of XC5397 was evaluated (*black*). The assays were conducted on the proteins treated (*bottom*) with and (*top*) without 1 mM EDTA. Each experiment was performed in triplicate, and error bars represent the standard deviation.

To investigate whether DrSOD exhibits gluconolactonase activity, sugar lactones, including D-(+)-glucono-1,5-lactone, L-(+)-gluonic acid γ-lactone, and D-(+)-ribono-1,4-lactone, were tested as substrates. Even in the absence of lactonases, lactones undergo non-enzymatic hydrolysis, acidifying the solution and reducing absorption at 405 nm due to the pH-sensitive indicator *p*-nitrophenol (21). For D-(+)-glucono-1,5-lactone, a gluconolactonase XC5397 significantly accelerated the decrease in absorption, an effect that was abolished upon the addition of EDTA, a calcium ion chelator, confirming its calcium-dependent lactonase activity (Fig. 7*A*, black circles) (21). In contrast, DrSOD, regardless of its copper and zinc ion binding status, caused only a slight acceleration in absorption reduction, indicating a lack of efficient enzymatic activity (Fig. 7*A*, blue and red circles). Neither of the individual domains of DrSOD exhibited the lactonase activity toward D-(+)-glucono-1,5-lactone (Fig. S5*A*). Consistent with previous findings (21), moreover, XC5397 showed no lactonase activity for L-(+)-gluonic acid γ-lactone and D-(+)-ribono-1,4-lactone, and DrSOD similarly did not exhibit significant activity toward these substrates over a longer time frame (∼17 h, Fig. S6, *A* and *B*).

To identify substrates for DrSOD, various γ-lactones were tested, including γ-valerolactone, γ-decanolactone, α-heptyl-γ-butyrolactone, α-acetyl-γ-butyrolactone, γ-crotonolactone, and 3-methyl-2(5H)-furanone, but DrSOD as well as XC5397 did not exhibit significant lactonase activity for these compounds (Fig. S6, *C*–*H*). Similarly, no obvious activity was confirmed with homoserine lactones, including D-(+)-homoserine lactone, L-(−)-homoserine lactone, N-carbobenzoxy-L-homoserine lactone, N-octanoyl-DL-homoserine lactone, and N-dodecanoyl-L-homoserine lactone (Fig. S7, *A*–*E*). Furthermore, tests with acyl homoserine lactones (AHLs), such as N-hexanoyl-L-homoserine lactone (C6-HSL) and N-decanoyl-L-homoserine lactone (C10-HSL), using a paper disk assay with *Chromobacterium violaceum*, which produces the purple pigment *violacein* in response to AHLs (22, 23), showed no hydrolysis by DrSOD and DrSOD^β-pro^, as violacein production remained unaffected (Fig. S8).

After testing those substrates without observing significant lactonase activity, we examined 2-coumaranone and 3,4-dihydrocoumarin, both commonly used in lactonase activity assays. As shown in Figure 7*B* (black circles), XC5397 hydrolyzed 2-coumaranone, leading to a significant drop in solution pH, confirming its lactonase activity. DrSOD also hydrolyzed 2-coumaranone and appeared to exhibit higher catalytic efficiency than XC5397 (Fig. 7*B*, red and blue circles); however, its overall efficiency remained relatively low, with an estimated *K*_m_ of approximately 110 mM and a *v*_max_ of 6.1 mM/min, though these values may not be accurate due to the weak enzymatic activity (Fig. S9). The lactonase activity of DrSOD was abolished by the addition of EDTA but remained unaffected by its copper and zinc ion binding status (Fig. 7*B*, red and blue circles). Furthermore, we confirmed that the lactonase activity of DrSOD toward 2-coumaranone was attributed to DrSOD^β-pro^ but not DrSOD^SOD^ (Fig. S5*B*). DrSOD, however, showed almost insignificant activity toward 3,4-dihydrocoumarin, which was efficiently hydrolyzed by XC5397 (Fig. 7*C*). Similarly, we confirmed that DrSOD^β-pro^ has no lactonase activity toward 3,4-dihydrocoumarin, suggesting that DrSOD^SOD^ does not contribute to substrate specificity (Fig. S5*C*). These findings demonstrate that DrSOD specifically hydrolyzes 2-coumaranone at the calcium ion bound in the DrSOD^β-pro^ domain, independent of the DrSOD^SOD^ domain.

Given the similarity of its calcium-binding site to that of DFPase (Fig. 4), it is essential to evaluate whether DrSOD exhibits phosphotriesterase activity toward known DFPase substrates, such as diisopropyl fluorophosphate and organophosphorus nerve agents like sarin (24). However, due to their high toxicity, those compounds could not be obtained. Notably, DFPase is highly specific for the substrates, and alternative compounds such as the readily accessible paraoxon are not hydrolyzed by DFPase (25). Nonetheless, we tested the potential phosphotriesterase activity of DrSOD using paraoxon as a surrogate substrate, and no enzymatic activity was detected (Fig. S10*A*), suggesting that DrSOD does not exhibit broad phosphotriesterase activity. Similarly, human serum paraoxonase 1, a calcium-dependent esterase with a six-bladed β-propeller structure, exhibits aryl esterase activity toward phenyl acetate; however, DrSOD did not show detectable hydrolytic activity toward phenyl acetate (Fig. S10*B*).

Similar to other six-bladed β-propeller lactonases, the mechanism governing substrate specificity in DrSOD remains unclear. Additionally, no consensus has been reached on the catalytic mechanism of β-propeller lactonases (26, 27, 28, 29); nonetheless, a water molecule that is either bound to Ca^2+^ (27, 28, 29) or located near the active site (26) is considered to play important roles in the lactonase activity. In some mechanisms, the water molecule becomes deprotonated by the Asp residue coordinating Ca^2+^, and the resulting hydroxide ion acts as a nucleophile attacking the carbonyl carbon of the substrate (28, 29). While such mechanistic diversity highlights the complexity of β-propeller lactonases, Asp381 and the water molecules at the Ca^2+^-binding site in DrSOD^β-pro^ (Fig. 4*A*) are expected to play critical roles in the hydrolysis of 2-coumaranone; further studies will be necessary to clarify the catalytic process in DrSOD^β-pro^.

### Cross-species occurrence of Cu/Zn-SOD and β-propeller fusion proteins

In the InterPro database, DrSOD^SOD^ is classified under SOD-like_Cu/Zn_dom/sf (IPR036423), SOD_Cu/Zn_/chaperone (IPR024134), and SOD_Cu_Zn_dom (IPR001424), while DrSOD^β-pro^ is categorized as 6-blade_b-propeller_TolB-like (IPR011042). Using those InterPro entries, we searched again for proteins containing both the Cu/Zn-SOD domain and the 6-bladed β-propeller domain. Out of the 56 hits obtained, 33 were identified as homologs of DrSOD across various species within the *Deinococcus* genus. Several large proteins were identified containing both Cu/Zn-SOD-like domain and 6-bladed β-propeller domain, which are, however, not directly connected with each other and also lack the residues necessary for superoxide dismutase and lactonase activity. Nonetheless, several other bacteria outside of *Deinococcus*, such as species in the *Acidovorax* genus, possessed proteins homologous to DrSOD. Furthermore, an eukaryotic rotifer, *Didymodactylos carnosus,* was found to harbor a protein homologous to DrSOD (UniProtKB accession number, A0A8S2GKV7); however, in this rotifer, the protein features an additional domain at its C-terminus, corresponding to an incomplete Cu/Zn-SOD domain lacking the metal binding site. The β-propeller domain appears to lack the ligands for Ca^2+^ binding, suggesting that the protein in *D. carnosus* likely serves a function distinct from that of DrSOD.

Since Cu/Zn-SOD is a type of immunoglobulin-like fold, it is also intriguing to note that several crystal structures have been deposited in the Protein Data Bank showing proteins with an immunoglobulin-like fold domain fused to a 6-bladed β-propeller domain, effectively replacing the Cu/Zn-SOD domain in a similar architectural context (PDB IDs: 3HRP, 3TC9, 3KYA). Those β-propeller domains are not limited to the 6-bladed form but also include 7-bladed variants (30), highlighting the structural diversity of this protein architecture.

### Possible significance of DrSOD in the physiology of *D. radiodurans*

DrSOD exhibits both superoxide dismutase activity and a unique lactonase activity specific to 2-coumaranone; however, the physiological relevance of those enzymatic functions in *D. radiodurans* remains unclear. While coumarin and its derivatives have been implicated in various biological processes—particularly in plant–microbe interactions such as iron acquisition, antimicrobial defense, and signaling (31)—DrSOD did not display lactonase activity toward either coumarin or 3,4-dihydrocoumarin (Figs. 7*C* and S6*I*). Instead, DrSOD exclusively hydrolyzes 2-coumaranone, a compound with no well-established biological function, apart from one report describing its nematicidal activity (32). While *D. radiodurans* failed to grow on TGY agar plates containing more than 5 mM 2-coumaranone (data not shown), supplementation with 1 mM 2-coumaranone had little effect on the growth of either the wild-type or ΔDrSOD strain under these conditions (Fig. S1*A*).

As noted in the Introduction, superoxide dismutase activity is crucial for *D. radiodurans* survival under ionizing radiation. In particular, manganese superoxide dismutase (MnSOD; DR1279), which exhibits much higher enzymatic activity than its *E. coli* counterpart (10), plays a key role in detoxifying the elevated intracellular superoxide generated by ionizing radiation. Inactivation of this enzyme increases cellular sensitivity to radiation stress (11). Additionally, *D. radiodurans* is characterized by its unusually high intracellular manganese ion concentration (∼4 mM), which further contributes to reactive oxygen species scavenging through Mn-phosphate complexes (9).

By contrast, Cu/Zn-superoxide dismutases in *D. radiodurans*—including DrSodC (DR1546), DR0644, and DrSOD (DRA0202)—are predicted to be localized in the periplasm. Among those, we found no significant contribution of DrSodC or DrSOD to UV resistance (Fig. S1*A*). In pathogenic bacteria, periplasmic SODs have been proposed to protect against host-derived oxidative bursts during infection; however, their physiological role in non-pathogenic Gram-negative bacteria such as *E. coli* and *Salmonella* remains ambiguous (2). An alternative function for Cu/Zn-SOD has been proposed in budding yeast, where cytoplasmic Cu/Zn-SOD is thought to chelate excess intracellular copper ions and mitigate copper-induced toxicity (33). *D. radiodurans* exhibited unchanged growth in TGY medium containing 0.1 mM CuSO_4_ (Fig. S1*C*) and growth suppression at 0.5 mM CuSO_4_ (data not shown), regardless of whether DrSodC, DrSOD, or both were deleted. These results suggest that DrSodC and DrSOD are unlikely to play a major role in protecting against toxicity of copper ions by chelating them. Taken together, while DrSOD possesses distinct enzymatic activities, its physiological function in *D. radiodurans* remains elusive and requires further investigation.

In summary, DrSOD was found to be expressed in *D. radiodurans* during the stationary phase, while its physiological role remains unclear. Nevertheless, DrSOD represents a unique type of metalloenzyme, integrating two functionally distinct catalytic domains within a single polypeptide. Each domain utilizes a different metal ion—a copper and zinc ion for DrSOD^SOD^ and a calcium ion for DrSOD^β-pro^—to independently drive superoxide dismutation and lactone hydrolysis, respectively. This dual-metal, dual-function arrangement distinguishes DrSOD from previously characterized enzymes, highlighting an unusual strategy for integrating multiple catalytic activities within a single protein scaffold. These findings provide new insights into the structural and functional diversity of metalloenzymes.

## Experimental procedures

### Preparation of recombinant proteins

The genome sequence encoding DrSOD is registered in the UniProt (34) as the accession number of Q9RYV4. The N-terminal region with the first 23 amino acids has been predicted to function as the signal peptide; therefore, the cDNA corresponding to the amino acid sequence from Ala24 to Phe462 in DrSOD was cloned into the multiple cloning site of a modified pET-15b plasmid vector (Novagen), in which the thrombin cleavage site was replaced with the HRV3C cleavage site. Similarly, the cDNAs of DrSOD^SOD^ (Ala24–Gly184), DrSOD^β-pro^ (Leu185–Phe462), and XC5397 from *Xanthomonas campertris pv. campestris*, which is a gluconolactonase registered as B0RN69 in the UniProt (34), were also cloned into the modified pET-15b. DrSOD^SOD^ has no Trp, making it difficult to determine the protein concentration; therefore, a Trp was added at the C-terminus of the protein.

DrSOD and DrSOD^SOD^ were overexpressed in *E. coli* SHuffle transformed with the corresponding plasmids. For the expression of DrSOD^β-pro^ and XC5397, *E. coli* BL21(DE3) was used. The expression of all those proteins was induced with 0.5 mM isopropyl-1-thio-β-D-galactopyranoside at 20^o^C overnight. For the purification of those proteins except DrSOD^SOD^, the same method was employed as described in the previous study (7): namely, the proteins were purified using a cOmplete His-Tag Purification Column (1 ml, Roche) followed by removal of the N-terminal 6× His tag with HRV3C and further purified by a gel filtration column (Cosmosil 5Diol-300-II, Nacalai Tesque) using a buffer containing 50 mM MOPS and 100 mM NaCl at pH 7.0 (MN buffer) as a running buffer. For the purification of DrSOD^SOD^, the metal ions bound at the protein was removed by the dialysis of the samples purified by the cOmplete His-Tag Purification Column against a buffer containing 50 mM sodium acetate, 100 mM NaCl, and 10 mM ethylenediaminetetraacetic acid (EDTA) at pH 4.0 followed by a buffer containing 100 mM Na-Pi, 100 mM NaCl, 5 mM EDTA at pH 7.0. The dialyzed proteins were treated with HRV3C and then purified by the gel filtration column. DrSOD and DrSOD^SOD^ prepared above were in the apo state, in which the copper and zinc content were confirmed to be less than 10% of the protein content using graphite furnace atomic absorption spectroscopy (AA-7000, Shimadzu). The protein concentration was determined spectroscopically using the molar extinction coefficient at 280 nm: 34,380 cm^-1^M^-1^ (DrSOD), 7115 cm^-1^M^-1^ (DrSOD^SOD^), 32,890 cm^-1^M^-1^ (DrSOD^β-pro^), and 60,960 cm^−1^M^−1^ (XC5397).

For the preparation of DrSOD and DrSOD^SOD^ in the holo form, the corresponding proteins in the apo state were first incubated with an equimolar amount of CuSO_4_ at 37 °C for 30 min and then with an equimolar amount of ZnSO_4_ at 37 °C for 30 min. Unbound copper and zinc ions were removed using centrifugal filtration to obtain DrSOD and DrSOD^SOD^ in the holo form. The copper and zinc content of those proteins were quantified using graphite furnace atomic absorption spectroscopy (AA-7000, Shimadzu), confirming that the metal content was approximately 100% relative to the protein concentration.

### Activity assays of DrSOD proteins

The assay for the superoxide dismutase activity was performed as described previously (17). An assay solution was prepared consisting of 100 mM sodium phosphate buffer (pH 8.0), 0.1 mM diethylenetriamine penta-acetic acid (DTPA), 0.1 mM hypoxanthine, 50 μM WST-1, and 10 μg/ml catalase. For each measurement, 200 μl of the assay solution was dispensed into the wells of a 96-well microplate, followed by the addition of 10 μl of protein solutions in 100 mM sodium phosphate buffer (pH 8.0) containing 0.1 mM DTPA. The mixtures were gently mixed to ensure homogeneity. Superoxide generation was then initiated by adding 5 μl of xanthine oxidase (Sigma, #X4500), pre-diluted 300-fold with water. The resulting reduction of WST-1 by superoxide was monitored by measuring the absorbance at 450 nm using a microplate reader (Epoch, BioTek). The percentage inhibition of WST-1 reduction was calculated and then plotted against the logarithm (base 10) of the protein amount (in pmol) added. The resulting inhibition curves were fitted with a sigmoidal function, and the IC_50_ value, which is defined as the amount of proteins required to achieve 50% inhibition, was used as an indicator of superoxide dismutase activity.

The lactonase activity was performed using *p*-nitrophenol as an indicator of the solution pH, which decreases in absorbance at 405 nm upon the hydrolysis of lactones (21). Briefly, 245 μl of 2 μM DrSOD proteins were prepared in a buffer at pH 7.0 containing 10 mM PIPES, 0.25 mM *p*-nitrophenol, and 20 mM CaCl_2_. The reaction was initiated by adding 5 μl of 100 mM substrates dissolved in DMSO, and absorbance at 405 nm was monitored every hour or every 10 min, depending on the experimental conditions, using a plate reader, Epoch (BioTek). For the estimation of *K*_m_ and *v*_max_, a calibration curve was constructed using acetic acid solutions of different concentrations, measuring their absorbance at 405 nm under the same assay conditions. The phosphotriesterase activity assay with Paraoxon and the aryl esterase activity assay with phenyl acetate were conducted using the same method. An assay for the degradation of acyl homoserine lactones was performed as follows; the purified protein solution (99 μl) was mixed with 1 μl of 1 mM N-hexanoyl-L-homoserine lactone (C6-HSL) or N-decanoyl-L-homoserine lactone (C10-HSL). After incubation at 30°C for 2 or 22 h, the remaining C6-HSL and C10-HSL were detected on LB agar plates containing *C. violaceum* CV026 (22) and VIR07 (23), respectively. Briefly, an overnight culture of CV026 or VIR07 was added to 25 ml of melted LB agar medium and solidified in a Petri dish. Paper discs (8-mm diameter; Advantec) were placed on LB agar plates containing CV026 or VIR07, and 20 μl of supernatants were applied to the discs. The plates were incubated overnight at 30 °C, and the presence of the remaining C6-HSL or C10-HSL was detected by the appearance of a purple pigment.

### Size exclusion chromatography with multi-angle static light scattering (SEC-MALS)

The molecular size of DrSOD, DrSOD^SOD^, and DrSOD^β-pro^ in solution were examined by SEC-MALS. The proteins in the concentration of 25 μM in the MN buffer with and without 5 mM EDTA were loaded on a gel filtration column (LW-803, SHODEX) fitted to an HPLC system (Shimadzu), and the absorbance change at 280 nm of the elution was monitored. The molecular size of the protein eluted from the column was determined by multi-angle light scattering using miniDAWN TREOS (WYATT Technology) connected on-line to the HPLC system.

#### Preparation of *D. radiodurans* lacking the gene coding DrSOD and DrSodC

*D. radiodurans* (NBRC 15346) was obtained from NBRC and cultured in TGY media at 30^o^C. The gene deletion in *D. radiodurans* was performed following the method described previously (35). The gene encoding DrSOD (*dra0202*) appears to form an operon with the gene encoding a PQQ-dependent sugar dehydrogenase (NCBI reference sequence: WP_027479768.1). Notably, the stop codon (TGA) of the PQQ-dependent sugar dehydrogenase overlaps with the start codon (ATG) of DrSOD at the underlined adenine; therefore, the following DNA cloning was performed so as to maintain the stop codon in the coding region of the PQQ-dependent sugar dehydrogenase. More precisely, a DNA construct for the deletion of *dra0202* was cloned into the pET15b vector (Novagen) by sequentially connecting the following regions: (1) the 5′ upstream region of *dra0202* (1000 bp; positions 255,643–256,643 in NZ_CP031501.1), (2) a 120 bp sequence located immediately upstream of the *katA* gene (*dr1998*) in *D. radiodurans* (positions 1,218,191–1,218,310 in NZ_CP031500.1), (3) the gene encoding chloramphenicol acetyltransferase (CAT) from pACYCDuet-1 (Novagen), and (4) the 3′ downstream region of *dra0202* (1000 bp; positions 258,029–259,028 in NZ_CP031501.1). For the deletion of *dr1546*, a DNA construct was cloned into the pET-15b vector (Novagen) by sequentially connecting the following regions: (1) the 5′ upstream region of *dra1546* (1000 bp; positions 765,818–764,819 in NZ_CP031500.1), (2) a 120 bp sequence located immediately upstream of the *katA* gene (*dr1998*) in *D. radiodurans* (positions 1,218,191–1,218,310 in NZ_CP031500.1), (3) the gene encoding aminoglycoside phosphotransferase (KanR) from pET28a (Novagen), and (4) the 3′ downstream region of *dr1546* (1000 bp; positions 764,269–763,270 in NZ_CP031500.1). The construct was amplified by PCR and then used to transform *D. radiodurans* on a TGY plate supplemented with 3 μg/ml chloramphenicol or 8 μg/ml kanamycin for the deletion of *dra0202* or *dr1546*, respectively, following a previously described method (36). Successful deletion of *dra0202* or *dr1546* by its replacement with the cDNA of *katA*:CAT or *katA*:KanR was confirmed by PCR, respectively.

### Analysis of endogenous DrSOD

A 5 ml culture of *D. radiodurans* NBRC 15346 cells in TGY medium was prepared at 0.01 of the optical density at 600 nm (OD_600_) in an L-shaped test tube and incubated at 30^o^C using a rocking incubator (TVS062CA, ADVANTEC) at 50 rpm. The incubator can automatically monitor the optical density at 660 nm of the cultures. The cells collected by centrifugation at 3000 *g* were resuspended in phosphate-buffered saline (PBS) containing 2% Triton X-100, 1 mM EDTA and Protease Inhibitor Cocktail (Nacalai) and was lysed by sonication using BIORUPTOR II (Sonicbio Co. Ltd). The total protein concentrations in the lysates were measured by Micro BCA Protein Assay Kit (Thermo Scientific). For the detection of DrSOD with Western blotting analysis, the lysates were prepared in the Laemmli sample buffer with 6.7% β-mercaptoethanol, separated in 12.5% polyacrylamide gels by SDS-PAGE, and then blotted on a PVDF membrane. After the membranes were blocked with 1% (w/v) skim milk in PBS containing 0.05% Tween-20 (PBS-T), the blots were probed with a polyclonal antibody (anti-DrSOD) that was raised in rabbits immunized with a peptide corresponding to Met102–His115 in DrSOD with an additional Cys at its N-terminus (Eurofins Genomics) and affinity-purified with the peptide conjugated with SulfoLink Coupling Resin (Thermo Fisher Scientific). The anti-DrSOD antibody was confirmed to recognize recombinant DrSOD proteins by Western blotting analysis.

### Effects of *dra0202* deletion on the stress resistance of *D. radiodurans*

*D. radiodurans* cells were cultured in TGY medium in L-shaped test tubes at 30°C using a rocking incubator (TVS062CA, ADVANTEC) at 50 rpm until reaching the stationary phase (OD_600_ ∼ 3.0). The cultured cells were harvested by centrifugation at 3000 *g* for 5 min at 20 °C and washed with sterile PBS. For the UV resistance assay, the washed cells were resuspended in PBS, placed on culture dishes without covers, and exposed to UV light at 254 nm using a UV Stratalinker 1800 (Stratagene). To assess resistance to mitomycin C (MMC), the cells were treated with 0.1 μg/ml MMC for 30 min at 30 °C. Following the stress treatments, the cells were serially diluted, spotted onto TGY agar plates. To examine effects of CuSO_4_ and 2-coumaranone on the growth of *D. radiodurans* cells, the cells resuspended in PBS were serially diluted and spotted onto TGY agar plates containing CuSO_4_ and 2-coumaranone, respectively. The agar plates were then incubated at 30 °C for 5 days to evaluate survival and growth.

### Crystallization

DrSOD samples were prepared by adding equimolar amounts of CuSO_4_ and ZnSO_4_ but without the addition of calcium ions, followed by concentration to 500 μM for crystallization screening. Initial crystallization screening was conducted using an automated crystallization robot (37), and the crystallization conditions were subsequently optimized. Crystals of DrSOD were obtained in a solution containing 20% (w/v) PEG 3350 and 0.2 M sodium malonate (pH 4.0) were subjected to further analysis. As described in the Results and discussion, however, those crystals did not bind zinc ions probably due to the low pH of the crystallization solution. To determine the structure of DrSOD with bound copper, zinc, and calcium ions, an alternative crystallization condition was selected utilizing a solution containing 1.6 M lithium sulfate and 0.1 M Tris (pH 8.0). Under these conditions, DrSOD prepared with equimolar amounts of CuSO_4_, ZnSO_4_, and CaCl_2_ was crystallized, and the resulting crystals obtained were subjected to further analysis.

### Data collection and structure calculation

X-ray diffraction data of calcium-free and calcium-bound DrSOD crystals were collected on beamlines BL-17A of KEK (Tsukuba) and BL44XU at SPring-8 (Harima), respectively. Crystals were quickly soaked in a cryoprotectant solution containing 20% (w/v) glycerol and flash-cooled in liquid nitrogen. Diffraction spots of both crystals were collected at 100 K on the EIGER X 16M detector (Dectris). The obtained diffraction data were processed by indexing and integrating the diffraction intensities using *XDS* (38) and further processed using *AIMLESS* (39) in the CCP4i2 program package. For calcium-bound DrSOD, the high-multiplicity dataset was used to observe anomalous scattering effects. The phase determinations were performed by molecular replacement using *MOLREP* (40) in the CCP4i suite, with the structure of DrSOD predicted by AlphaFold2 as a search model. Subsequently, iterative manual model correction using *Coot* (41) and structure refinement using *Refmac* (42) or *Phenix.refine* (43) were performed. The data collection and refinement statistics are summarized in Table S1.

## Data availability

The data that support the findings in this study are available upon request.

## Supporting information

This article contains supporting information.

## Conflict of interest

The authors declare that they have no conflicts of interest with the contents of this article.
