# Supplementary material for: Cu/Zn-superoxide dismutase naturally fused with a β-propeller lactonase in Deinococcus radiodurans
Source: J Biol Chem. 2025 Jul 18;301(8):110499. doi: 10.1016/j.jbc.2025.110499 (PMC12362111; doi:10.1016/j.jbc.2025.110499)
Supplement: Supplementary materials [file mmc1.pdf]

Table S1 Data collection, reduction and refinement statistics on the analysis of DrSOD crystals

	**calcium-free DrSOD**	**calcium-bound DrSOD** Native	**calcium-bound DrSOD** Zn peak
**Data collection**			
Wavelength (Å)	1.282	0.900	1.330
Resolution range	39.23 - 1.800 (1.864 - 1.800)	36.78 - 1.900 (1.968 - 1.900)	47.17 - 2.50 (2.59 - 2.50)
Space group	*P*43	*P*41	*P*41
Unit cell (Å)	*a* = 83.07, *b* = 83.07, *c* = 119.53	*a* = 133.28, *b* = 133.28, *c* = 76.52	*a* = 133.43, *b* = 133.43, *c* = 76.59
Total reflections	147719 (14551)	817941 (84051)	361949 (36586)
Unique reflections	73916 (7282)	105657 (10494)	46849 (4578)
Multiplicity	2.0 (2.0)	7.7 (8.0)	7.7 (8.0)
Completeness (%)	98.67 (97.25)	99.96 (99.99)	100.0 (100.0)
$I/\sigma(I)$	13.68 (2.83)	24.82 (1.99)	12.3 (1.6)
Wilson *B*-factor (Å^2)	26.08	40.18	41.46
R_{merge}	0.031 (0.262)	0.043 (0.939)	0.115 (1.210)
R_{pim}	0.031 (0.262)	0.016 (0.353)	0.044 (0.456)
$CC_{1/2}$	0.997 (0.810)	1.000 (0.776)	0.998 (0.680)
**Refinement**			
Reflections used in refinement	73902 (7277)	105640 (10494)	
Reflections used for R_{free}	3664 (341)	5285 (554)	
R_{work}	0.1632 (0.2321)	0.1958 (0.3197)	
R_{free}	0.1964 (0.2945)	0.2198 (0.3398)	
Number of atoms	6463	6872	
macromolecules	5907	6515	
ligands	8	43	
solvent	548	314	
RMS (bonds) (Å)	0.009	0.007	
RMS (angles) (deg.)	1.06	0.87	
Ramachandran plot			
favored (%)	97.55	95.63	
allowed (%)	2.45	4.37	
outliers (%)	0	0	
Rotamer outliers (%)	1.3	0.88	
Average *B*-factor (Å^2)	33.57	44.69	
macromolecules (Å^2)	33.24	44.66	
ligands (Å^2)	51.4	49.54	
solvent (Å^2)	36.88	44.59	

Values in parentheses are for the highest resolution shell.

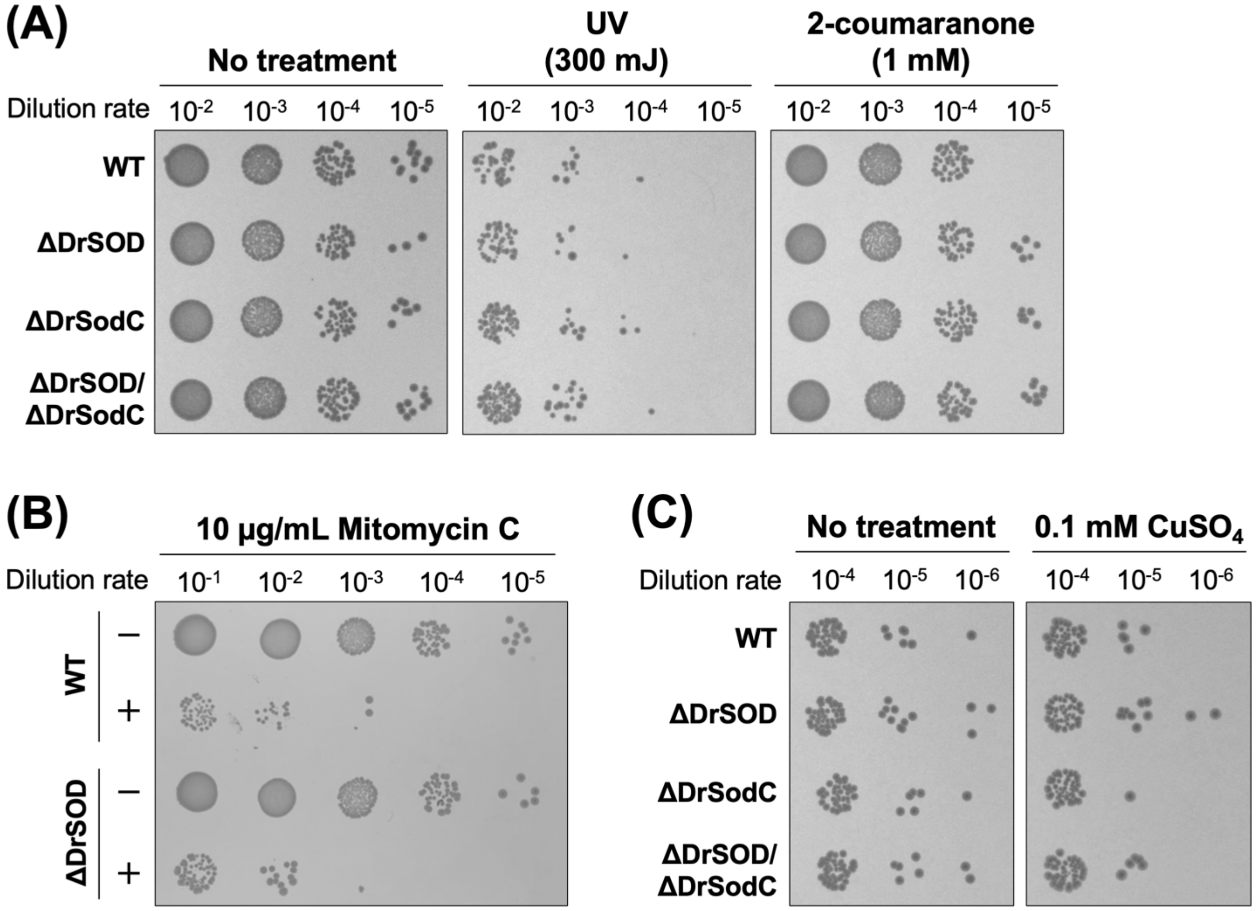

Figure S1 **No significant role of DrSOD in the growth of *D. radiodurans* under stress conditions** **(A)** As described in Experimental Procedures, *D. radiodurans* strains (WT, ΔDrSOD, ΔDrSodC, ΔDrSOD/ΔDrSodC) were cultured to the stationary phase in TGY medium, washed with PBS, and subjected to UV irradiation at 254 nm (300 mJ). Serial dilutions of untreated (left) and UV-treated (middle) cells were spotted onto TGY agar plates. To examine effects of 2-coumaranone, untreated cells washed with PBS were spotted onto TGY agar plates supplemented with 1 mM 2-coumaranone (right). **(B)** *D. radiodurans* cells washed with PBS were treated with 10 μg/mL mitomycin C, and serial dilutions of treated (+) and untreated (-) cells were spotted onto TGY agar plates. **(C)** Serial dilutions of *D. radiodurans* cells washed with PBS were spotted onto TGY agar plates without (left) or with (right) 0.1 mM $CuSO_4$.

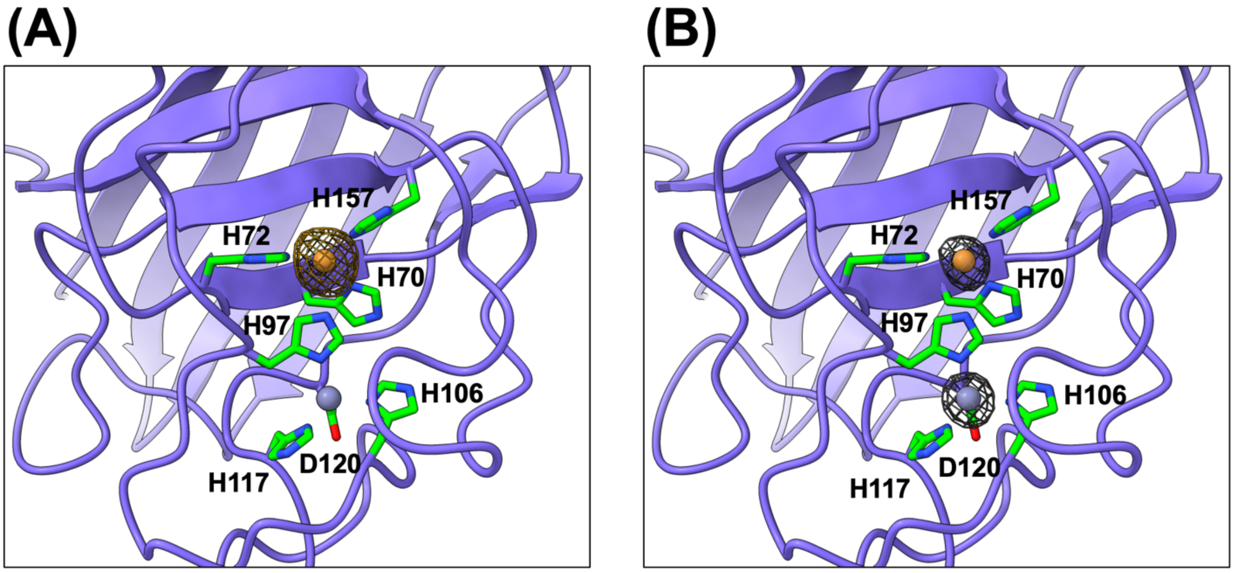

Figure S2 Characterization of metal binding at distinct sites in DrSOD^{SOD} Anomalous scattering difference maps were contoured at the 10σ level using diffraction data measured at wavelengths of **(A)** 1.330 Å and **(B)** 0.900 Å, and are shown as mesh around the metal-binding sites in DrSOD^{SOD}. Copper and zinc atoms are depicted as orange and cornflower blue spheres, respectively, with their coordinating ligands represented as stick models. At a wavelength of 0.900 Å, where anomalous scattering occurs for both copper and zinc, significant difference density was observed at both the copper- and zinc-binding sites. In contrast, at 1.330 Å, where anomalous scattering is observed only for copper but not for zinc, the difference density at the zinc-binding site was markedly reduced. These results confirm the assignment of copper and zinc ions at the distinct metal-binding sites, as indicated in the figure.

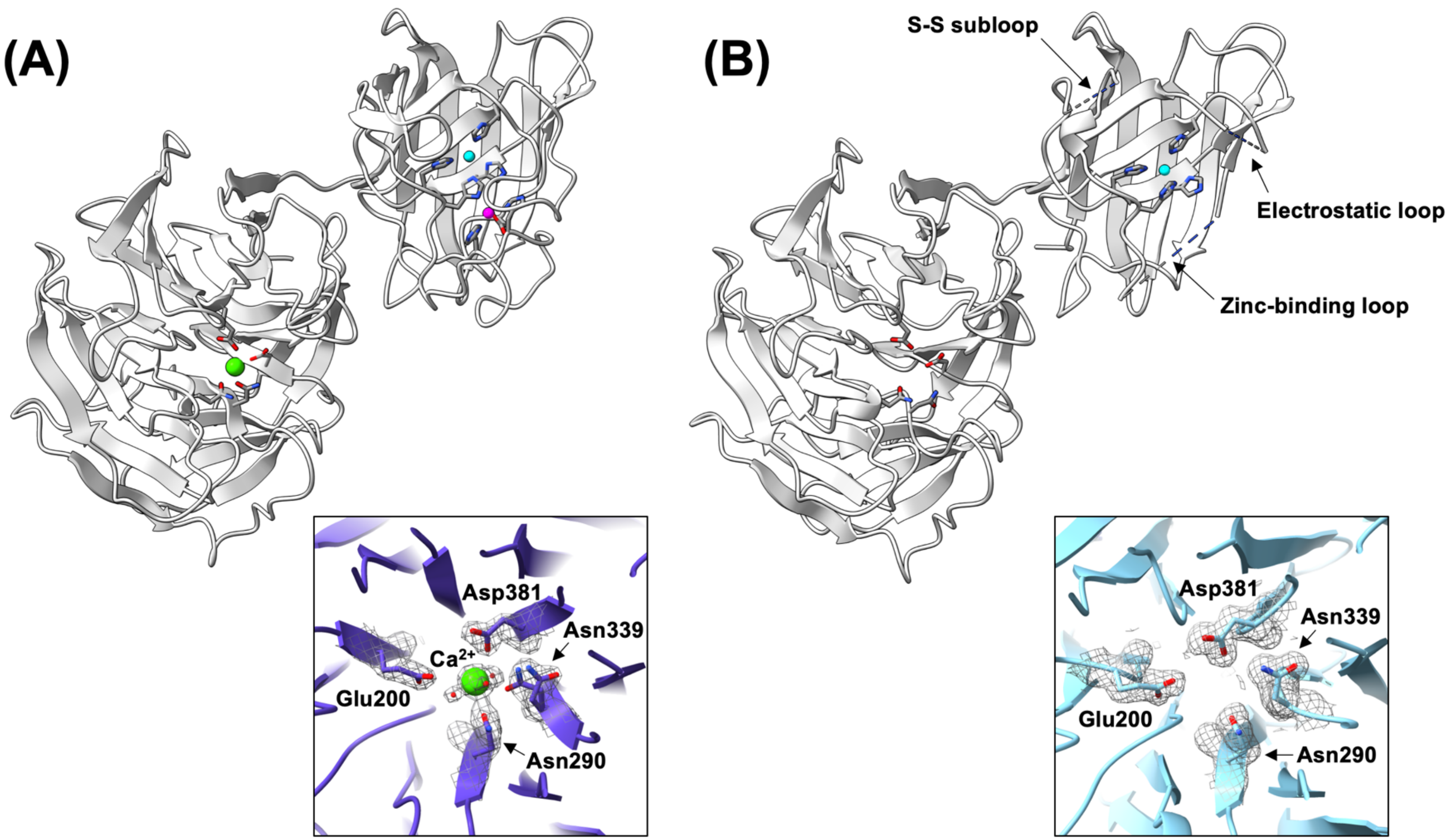

Figure S3 Structural comparison between calcium-bound and calcium-free form of DrSOD The overall structures of **(A)** calcium-bound DrSOD, as shown in Figure 3, and **(B)** calcium-free DrSOD are presented. Copper, zinc, and calcium ions are depicted as cyan, magenta, and light green spheres, respectively, with their coordinating ligands represented as stick models. Magnified views of the calcium-binding sites in both the calcium-bound and calcium-free forms are shown at the bottom of each panel. 2Fo-Fc maps, contoured at the 1.5σ level, are displayed as mesh around the calcium-binding site in both forms.

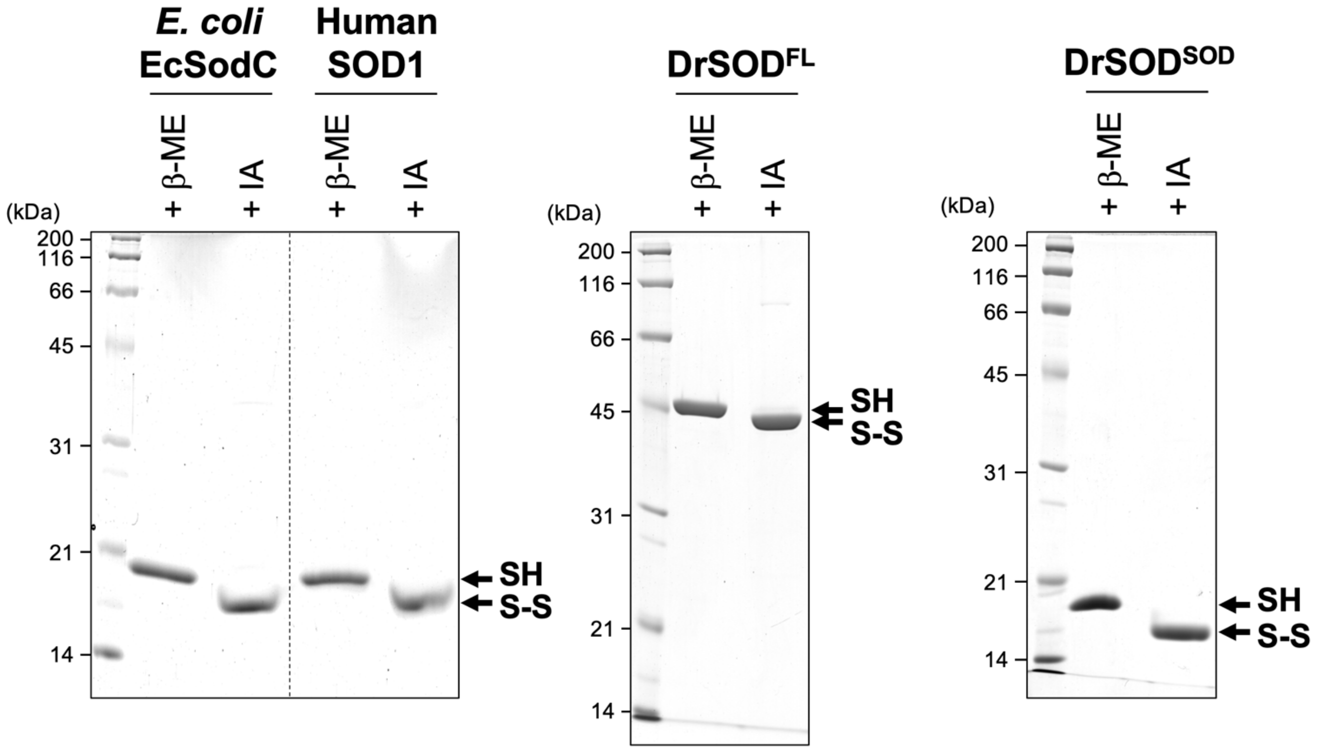

Figure S4 Confirmation of the formation of the conserved disulfide bond in Cu/Zn-SOD samples including DrSOD Cu/Zn-SOD proteins (~2 µg) were first treated with iodoacetamide (IA) to alkylate free thiol groups, if present, then mixed with Laemmli buffer lacking reducing agents, boiled, and subjected to SDS-PAGE using 12.5% gels for DrSOD^{FL} and 15% gels for EcSodC, SOD1, and DrSOD^{SOD} (indicated as +IA). In parallel, the same protein samples were treated with β-mercaptoethanol (β-ME) to reduce disulfide bonds, mixed with Laemmli buffer, boiled, and analyzed by SDS-PAGE using the same gels (indicated as +β-ME). A slight decrease in electrophoretic mobility in the samples treated with β-ME (indicated as SH) compared to those with IA (indicated as S-S) supports the presence of the conserved intramolecular disulfide bond in the Cu/Zn-SOD samples used in this study.

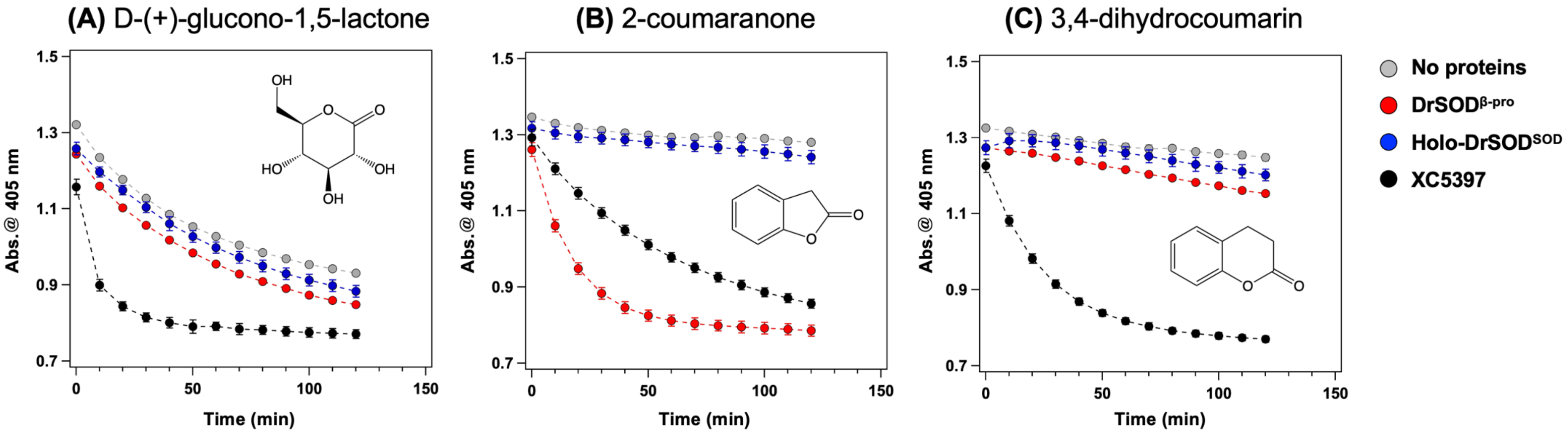

Figure S5 **Assay of lactonase activity of DrSOD^{SOD} and DrSOD$^{\beta\text{-pro}}$** The absorbance change at 405 nm, reflecting the pH changes of the solution, was monitored at an interval of 10 min using **(A)** D-(+)-glucono-1,5-lactone, **(B)** 2-coumaranone, and **(C)** 3,4-dihydrocoumarin as substrates. Lactonase activity was assessed for (red) DrSOD$^{\beta\text{-pro}}$ with a calcium ion, and (blue) holo-DrSOD^{SOD} containing a copper and zinc ion. A negative control without any protein was also included (gray). For comparison, the lactonase activity of XC5397 was evaluated (black). Each experiment was performed in triplicate, and error bars represent the standard deviation.

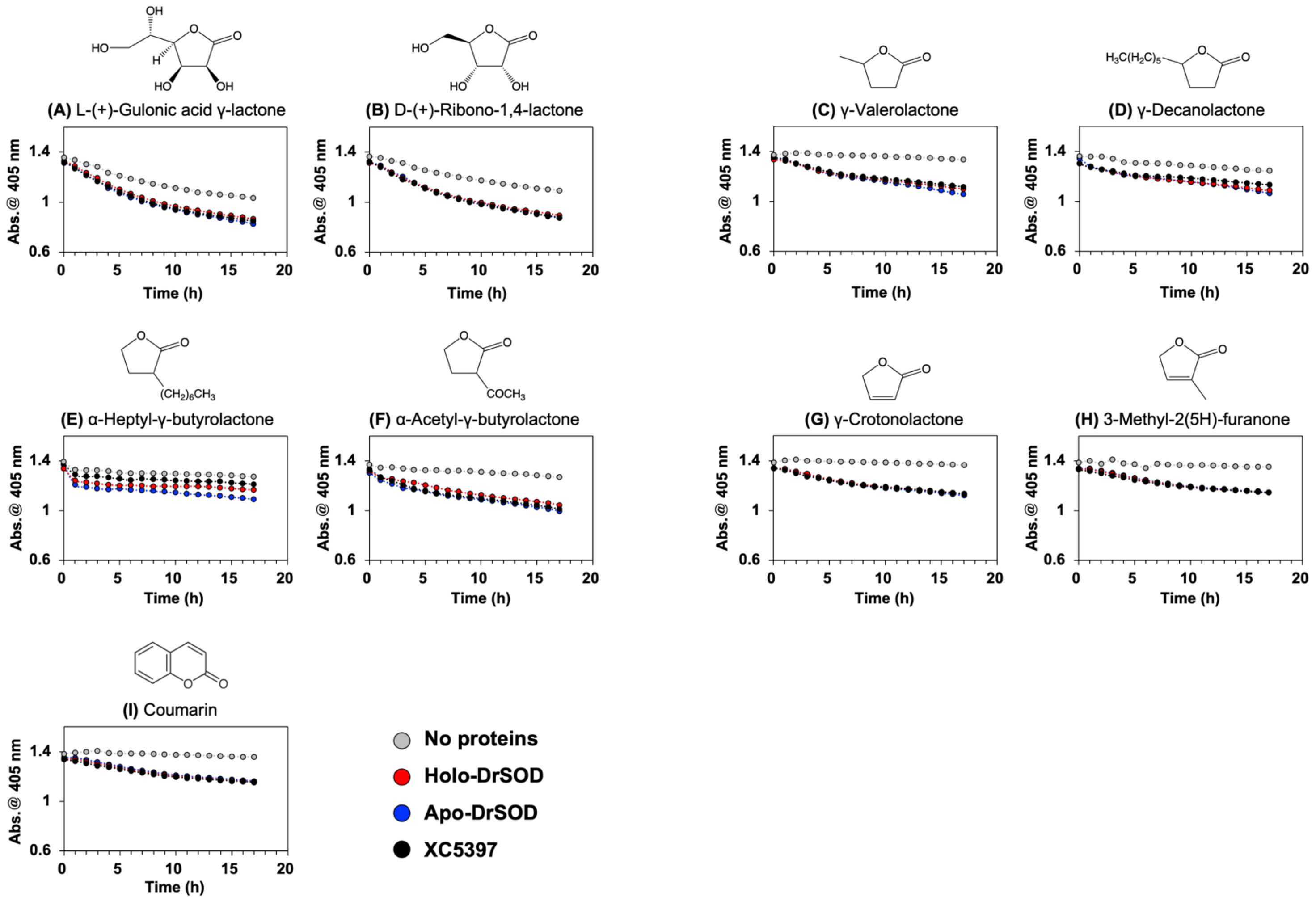

Figure S6 **Assay of lactonase activity of DrSOD for lactones** The absorbance change at 405 nm, reflecting the pH changes of the solution, was monitored at an interval of 1 hour using the lactones indicated as substrates. Lactonase activity was assessed for (red) holo-DrSOD with a copper, zinc, and calcium ion, and (blue) apo-DrSOD containing only a calcium without a copper and zinc ion. A negative control without any protein was also included (gray). For comparison, the lactonase activity of XC5397 was evaluated (black).

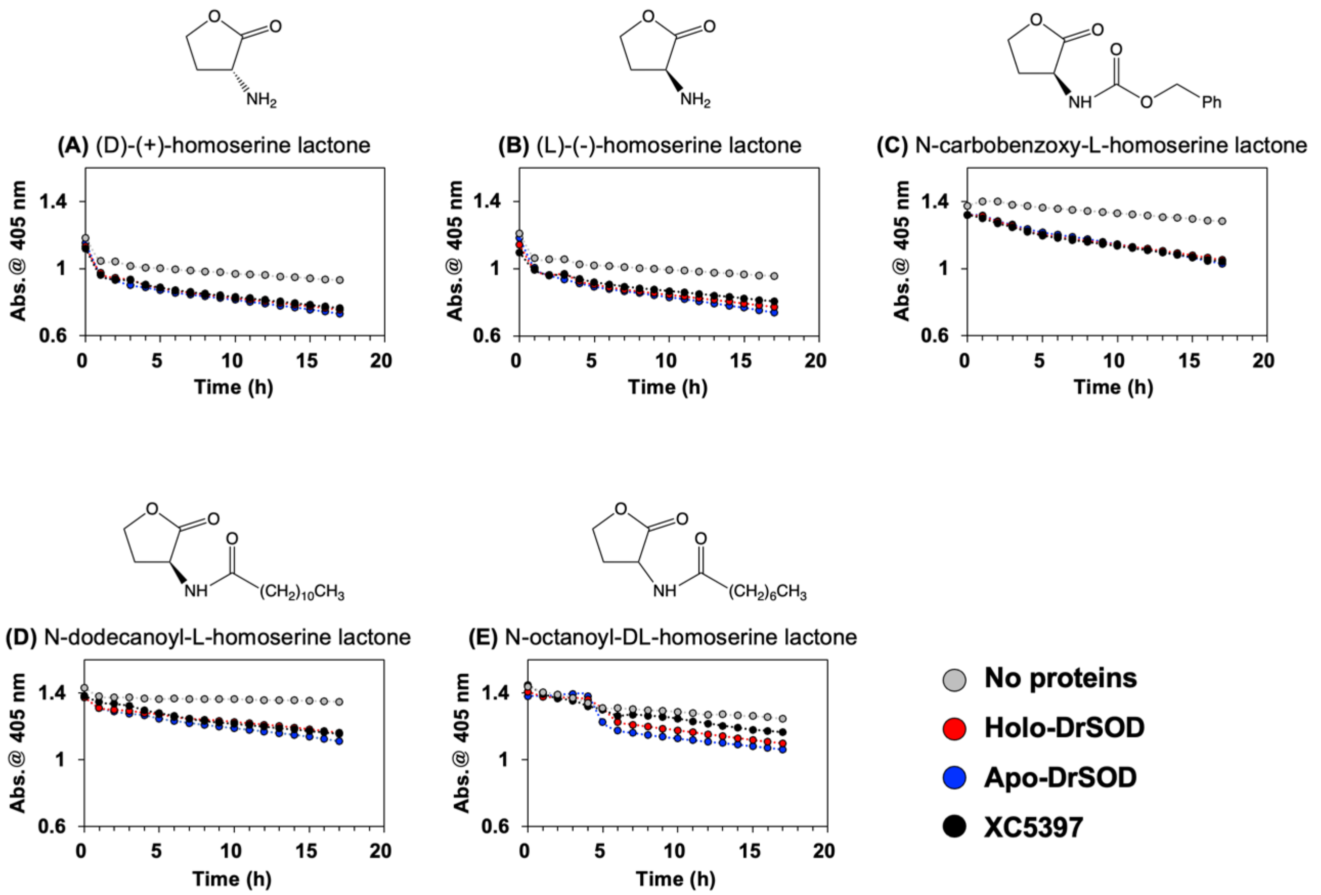

Figure S7 **Assay of lactonase activity of DrSOD for homoserine lactones** The absorbance change at 405 nm, reflecting the pH changes of the solution, was monitored at an interval of 1 hour using the homoserine lactones indicated as substrates. Lactonase activity was assessed for (red) holo-DrSOD with a copper, zinc, and calcium ion, and (blue) apo-DrSOD containing only a calcium without a copper and zinc ion. A negative control without any protein was also included (gray). For comparison, the lactonase activity of XC5397 was evaluated (black).

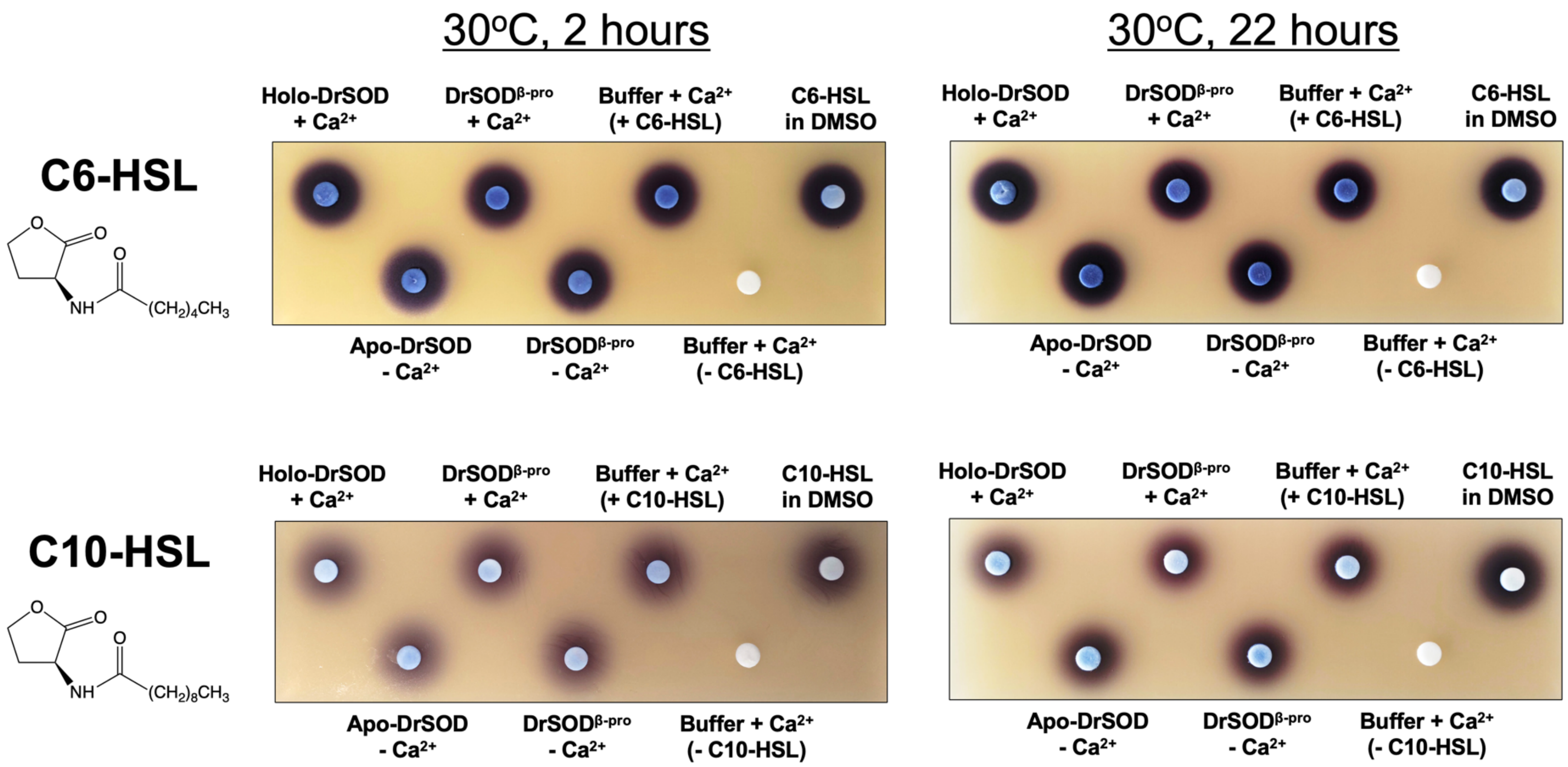

Figure S8 **Paper disk assay for the hydrolysis of acyl homoserine lactones** This assay detects the production of violacein, a purple pigment, when homoserine lactones are present in the samples, specifically C6-HSL (top) and C10-HSL (bottom). As shown, C6-HSL and C10-HSL were incubated with holo-DrSOD with calcium ions, apo-DrSOD without calcium ions, DrSOD$^{\beta\text{-pro}}$ with and without calcium ions at 30°C for (left) 2 hours and (right) 22 hours. The mixtures were then adsorbed onto filter papers and placed on LB agar plates containing *Chromobacterium violaceum* CV026 and VIR07, which produce violacein in response to C6-HSL and C10-HSL, respectively. As controls, the MN buffer with and without calcium ions as well as C6-HSL/C10-HSL dissolved in DMSO were tested.

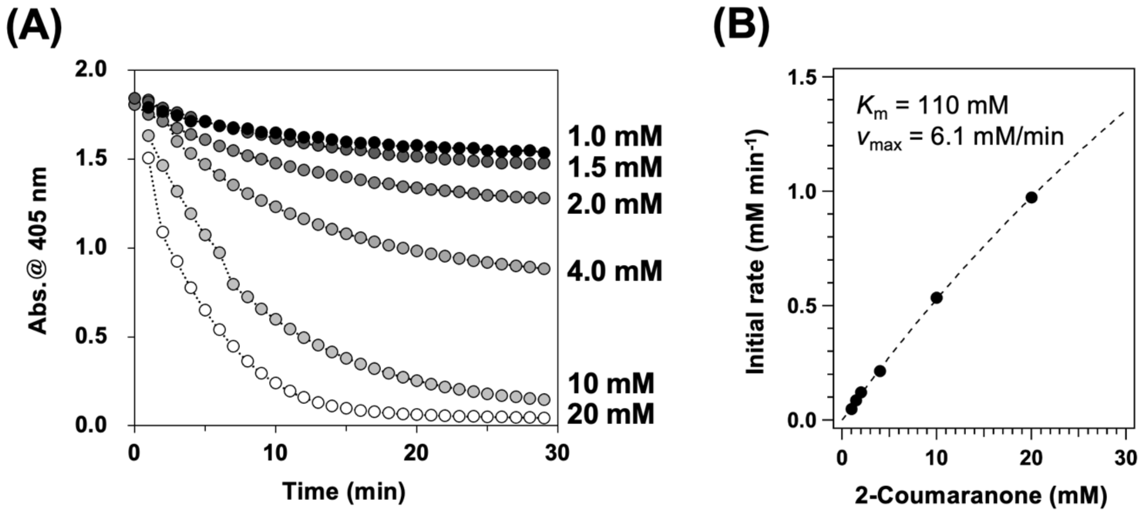

Figure S9 Estimation of enzyme kinetic parameters of DrSOD for the hydrolysis of 2-coumaranone **(A)** Holo-DrSOD containing a copper, zinc, and calcium ion at a concentration of 2 μM was mixed with 2-coumaranone at varying concentrations ranging from 1 to 20 mM (as indicated in the figure). The absorbance change at 405 nm, reflecting the pH changes of the solution upon lactone hydrolysis, was monitored at an interval of 1 min. **(B)** The initial hydrolysis rate of 2-coumaranone was calculated from the decrease in the absorbance at 405 nm during the first minute. This change was converted to the concentration of the corresponding hydroxy acid using a calibration curve established with acetic acid. The initial rates were then plotted against the concentrations of 2-coumaranone and fitted to the Michaelis-Menten equation to estimate the kinetic parameters K_m and v_{max}.

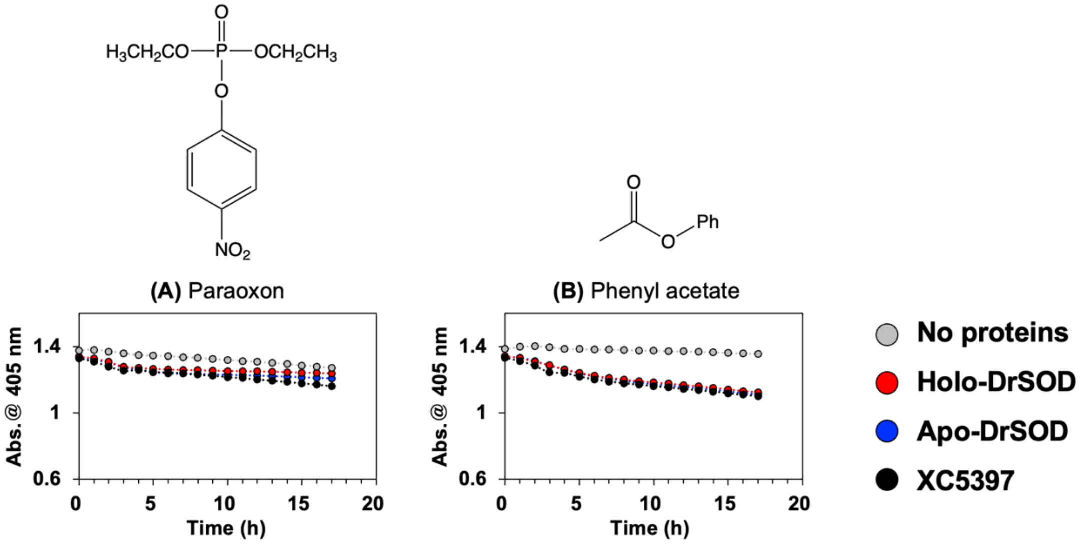

Figure S10 Assay of phosphotriesterase and aryl esterase activity of DrSOD The absorbance change at 405 nm, reflecting the pH changes of the solution, was monitored at an interval of 1 hour using **(A)** paraoxon and **(B)** phenyl acetate as the substrates for phosphotriesterase and aryl esterase activity, respectively. Those activities were assessed for (red) holo-DrSOD with a copper, zinc, and calcium ion, and (blue) apo-DrSOD containing only a calcium without a copper and zinc ion. A negative control without any protein was also included (gray). For comparison, the activities of XC5397 were evaluated (black).
